# Calibrated EWMA estimators for time-scaled surveys with diverse applications

**DOI:** 10.1016/j.heliyon.2024.e31030

**Published:** 2024-05-14

**Authors:** Abdullah Mohammed Alomair, Soofia Iftikhar

**Affiliations:** a Department of Quantitative Methods, School of Business, King Faisal University, Al-Ahsa 31982, Saudi Arabia; b Department of Statistics, Shaheed Benazir Bhutto Women University Peshawar, KP, Pakistan

**Keywords:** Time-scaled surveys, Stratified random sampling, Auxiliary information, Exponentially weighted moving average, Memory type mean estimators, Calibration

## Abstract

Recently, several memory-type mean estimators (including ratio, product, and logarithmic) have been developed. These estimators rely on exponentially weighted moving average (EWMA), which incorporate both historical and present sample data. In this article, we propose EWMA type calibrated estimators under single and double stratified random sampling ($S_{t}RS$). Because calibration method enhances the estimates by modifying the stratification weight, taking advantage of supplementary information. To evaluate the performance of estimators, various real-world time-scaled data sets pertaining to stock market and weather are taken into account. Additionally, we also conduct a simulation study using a bivariate symmetric data set. The numerical results show the superiority of proposed estimators $\left({\overset{¯}{y}}_{TM},{\overset{¯}{y}}_{TaM}\right)$ over the adapted ones $\left({\overset{¯}{y}}_{PM},{\overset{¯}{y}}_{PaM}\right)$.

## 1 Introduction

Many surveys are conducted regularly with specific time intervals. For example, Pakistan Social and Living Standards Measurement (PSLM) surveys are designed to furnish social and economic indicators at both district and provincial levels, alternating every year. Additionally, the government of Pakistan, through Bureau of Statistics, conducts the Labor Force Survey annually. Likewise, various other surveys, including those related to health, economics, agriculture, and more, are carried out at regular intervals worldwide. Noor-ul-Amin [1] has devised a methodology for parameter estimation, aimed at enhancing the outcomes of the aforementioned time-scaled surveys.

Initially, Noor-ul-Amin [1] aimed to estimate mean of study variable related to time scaled surveys using EWMA statistic. Roberts [2] holds the distinction of pioneering the introduction of the EWMA statistic. The formula of EWMA statistic is

(1) $$Z_{t}=\lambda \overset{¯}{y}+(1-\lambda )Z_{t-1}$$

In equation (1), $\overset{¯}{y}$ is the current sample mean, and *λ* represents the smoothing constant. In this context, ‘t’ represents the sample index, and the notation $Z_{t-1}$ pertains to earlier observations.

The significance of incorporating auxiliary information to enhance the effectiveness of estimators has been widely acknowledged. As a result, in recent years, numerous authors have proposed a plethora of ratio, product, regression, and exponential-type estimators that make use of diverse auxiliary information based on the current sample. Such as, Zaman [3] developed robust regression type mean estimators. Zaman and Bulut [4] provided the concept of robust covariance matrices in ratio and regression type mean estimators. Ali et al. [5] extended Zaman and Bulut [4] concept for sensitive variables using different scrambled response models. Koyuncu and Al-Omari [6] defined robust regression type mean estimators under different ranked set sampling schemes. Koc et al. [7] utilized Poisson regression coefficient and suggested ratio type regression mean estimators under double sampling. Bhushan et al. [8] attempted to develop efficient difference and ratio type estimators for the case of missing observations using imputation methods under ranked Set Sampling. For more details see references [9], [10], [11]. All these mean type estimators based on current sample information.

Utilizing the information from both current and previous samples i.e. $\left({\overset{¯}{y}}_{t},{\overset{¯}{y}}_{t-1},{\overset{¯}{y}}_{t-2}\right)$ for respective times t,t−1,t−2, and so on, can further enhance the efficiency of the estimator. This takes on heightened significance in scenarios where surveys are routinely conducted at fixed time intervals, like monthly or yearly surveys. Noor-ul-Amin [1], [12] defined EWMA and Hybrid EWMA type mean estimators using auxiliary information. Subsequently, Aslam et al. [13], [14] defined same kind of mean estimators under ranked set sampling. Moving on to time-based surveys, interested readers, see [15], [16], [17] and [18]. Bhushan et al. [19], [20] emphasize the importance of investigating logarithmic type estimators.

However, to the best of our knowledge, there has been no investigation into calibration-based EWMA mean estimators under ($S_{t}RS$). Calibration has a number of real life applications. For instance, calibration is essential for maintaining the accuracy and reliability of critical measurements in airplanes, such as altimeters and airspeed indicators, ensuring safe operation during flight. It involves using precision pressure sensors and calibrators to verify and adjust these devices, as well as calibrating on-engine sensors to guarantee accurate engine performance. Calibration plays an important role in weather stations as it certifies the accuracy and reliability of measurements, such as barometric pressure, which are important for creating isobars and assessing wind conditions. By using precision transducers and indicators, calibration allows the generation of reliable weather reports and predictions of significant weather situations based on local ambient pressure fluctuations. Calibration is indispensable in various areas of a hospital such as encompassing hospital bed sensors, pressure chambers, clean rooms, and breathing apparatus. It verifies the precise measurement and control of barometric pressure in pressure chambers and clean rooms to prevent the transmission of germs, while also facilitating optimal patient positioning and weight balance through calibration of hospital bed sensors. Moreover, calibration is crucial for maintaining the safety and performance of pressure sensors used in breathing apparatus, such as ventilators and respirators, which are vital for accurate measurement of oxygen flow and patient well-being. These are just few of the many everyday applications of calibration. Calibration can also be used to improve parameter estimates. Therefore, the objective of this study is to introduce memory type calibrated estimators that leverage both historical and present sample data, under $S_{t}RS$. To accomplish this goal, the study incorporates the use of the EWMA statistic.

The paper contains several sections that contribute significantly to the research. In Section 2, calibration technique is adapted for mean estimation. Which is a new approach in the context of memory-type mean estimation. Building on this, Section 3 introduces the proposed memory type calibrated estimator, which appears to offer further advancements and improvements over existing or adapted ones, promising better results in various applications. Additionally, Section 4 presents adapted and proposed estimators under double stratified random Sampling technique and making it an intriguing part of the paper. Section 5 contains a simulation study with diverse applications. This simulation study likely provides concrete evidence of the effectiveness and robustness of the calibrated estimators under single and double $S_{t}RS$. Finally, Section 6 serves as an essential part of the paper, summarizing the main findings and implications of the research.

## 2 Adapted memory type calibrated estimator

EWMA is a quantitative or statistical metric employed for modeling and characterizing time series data. References [21], [22] highlight some notable applications of EWMA in the detection of cyber attacks on computer networks. The construction of the moving average involves assigning lower weights to past data as more data points are included. It's worth noting that estimators linked to average and variability can also benefit from improvements by integrating auxiliary data.

Utilizing auxiliary information holds the promise of significantly improving mean estimators. In diverse real-world situations, a linear correlation between the variable of interest, *Y*, and an auxiliary variable, *X*, has been evident, as demonstrated in studies by Abbasi et al. [23] and Koc and Koc [24]. Some examples of correlation include depression vs. suicide (Johnson et al. [25]) and body mass index (BMI) vs. total cholesterol (Schroder et al. [26]). These examples serve to illustrate how auxiliary variables can supply valuable information and enhance the precision of mean estimation.

Calibration estimation is a fundamental technique focused on improving initial values by reducing a selected measure of inconsistency while integrating supplementary information. Developing new calibration weights depends on two critical factors: 1) the distance metric, and 2) constraints. These elements provide the basis for creating enhanced calibration weights. Given the robust correlation between the variables, it is anticipated that effective weights applied to the auxiliary variable would also be effective for the research variable. Drawing from the groundbreaking contributions of Deville and Sarndal [27], numerous researchers have delved into calibration estimation, employing various calibration constraints in the field of survey sampling, as documented in references [28], [29], [30].

Suppose we have *L* strata and $\left(X_{\vartheta },Y_{\vartheta }\right)$ be the study and auxiliary variables and $\left(Q_{\vartheta_{t}},Z_{\vartheta_{t}}\right)$ be there respective EWMA statistics in $\vartheta^{th}$ stratum as follows

$$Z_{\vartheta_{t}}=\lambda {\overset{¯}{y}}_{\vartheta }+(1-\lambda )Z_{\vartheta_{\left(t-1\right)}}$$

$$Q_{\vartheta_{t}}=\lambda {\overset{¯}{x}}_{\vartheta }+(1-\lambda )Q_{\vartheta_{\left(t-1\right)}}$$

Drawing inspiration from the significant studies discussed in previous lines, the memory type calibrated mean estimator ${\overset{¯}{y}}_{PM}$ using EWMA under $S_{t}RS$ framework is given below in equation (2):

(2) $${\overset{¯}{y}}_{PM}=\sum_{\vartheta =1}^{L}\Psi_{\vartheta }Z_{\vartheta_{t}}$$

subject to the constraints

(3) $$\sum_{\vartheta =1}^{L}\Psi_{\vartheta }Q_{\vartheta_{t}}=\sum_{\vartheta =1}^{L}W_{\vartheta }{\overset{¯}{X}}_{\vartheta }$$

(4) $$\sum_{\vartheta =1}^{L}\Psi_{\vartheta }c_{x_{\vartheta }}=\sum_{\vartheta =1}^{L}W_{\vartheta }C_{x_{\vartheta }}$$

(5) $$\sum_{\vartheta =1}^{L}\Psi_{\vartheta }=\sum_{\vartheta =1}^{L}W_{\vartheta }$$

where $W_{\vartheta }=\frac{N_{\vartheta }}{N}$ is the stratified weight, $\Psi_{\vartheta }$ is calibrated weight, ${\overset{¯}{X}}_{\vartheta }$ is the population mean, $\left(C_{x_{\vartheta }},c_{x_{\vartheta }}\right)$ are the respective population and sample coefficient of variations of auxiliary variable in $\vartheta^{th}$ stratum. For finding the calibrated weight $\Psi_{\vartheta }$, we will use the chi-square distance function $\sum_{\vartheta =1}^{L}\frac{{\left(\Psi_{\vartheta }-W_{\vartheta }\right)}^{2}}{\tau_{\vartheta }W_{\vartheta }}$ and the calibration constraints defined in the equations (3), (4) and (5).

Now, defining the Lagrange function with its multipliers $\eta_{1(\vartheta )}$, $\eta_{2(\vartheta )}$ and $\eta_{3(\vartheta )}$

$$\Delta_{\left(a\right)}=\sum_{\vartheta =1}^{L}\frac{{\left(\Psi_{\vartheta }-W_{\vartheta }\right)}^{2}}{\tau_{\vartheta }W_{\vartheta }}-2\eta_{1(\vartheta )}\left[\;\sum_{\vartheta =1}^{L}\Psi_{\vartheta }Q_{\vartheta_{t}}-\sum_{\vartheta =1}^{L}W_{\vartheta }{\overset{¯}{X}}_{\vartheta }\;\right]-2\eta_{2(\vartheta )}\left[\sum_{\vartheta =1}^{L}\Psi_{\vartheta }c_{x_{\vartheta }}-\sum_{\vartheta =1}^{L}W_{\vartheta }C_{x_{\vartheta }}\right]-2\eta_{3(\vartheta )}\left[\sum_{\vartheta =1}^{L}\Psi_{\vartheta }-\sum_{\vartheta =1}^{L}W_{\vartheta }\right].$$

Note that $\tau_{\vartheta }$ be 1 or reciprocal of any known characteristics of supplementary variable. By calculating $\frac{\delta \Delta_{\left(a\right)}}{\delta \Psi_{\vartheta }}=0$, we get

(6) $$\Psi_{\vartheta }=W_{\vartheta }+\tau_{\vartheta }W_{\vartheta }\left[\eta_{1(\vartheta )}Q_{\vartheta_{t}}+\eta_{2(\vartheta )}c_{x_{\vartheta }}+\eta_{3(\vartheta )}\right],$$

equation Substituting equation (6) in equation (3), equation (4), and equation (5), respectively, and get a system of equations containing 3 equations as

(7) $$G_{\left(3\times 3\right)}\eta_{\left(3\times 1\right)}=F_{\left(3\times 1\right)},$$

where

$$\eta_{\left(3\times 1\right)}=\left[\begin{array}{c} \eta_{1(\vartheta )}\\ \eta_{2(\vartheta )}\\ \eta_{3(\vartheta )} \end{array}\right],$$

$$F_{\left(3\times 1\right)}=\left[\begin{array}{c} \sum_{\vartheta =1}^{L}W_{\vartheta }\left({\overset{¯}{X}}_{\vartheta }-Q_{\vartheta_{t}}\right)\\ \sum_{\vartheta =1}^{L}W_{\vartheta }\left(C_{x_{\vartheta }}-c_{x_{\vartheta }}\right)\\ 0 \end{array}\right],$$

$$G_{\left(3\times 3\right)}=\left[\begin{array}{ccc} \left(\sum_{\vartheta =1}^{L}\tau_{\vartheta }W_{\vartheta }Q_{\vartheta_{t}}^{2}\right)\; & \left(\sum_{\vartheta =1}^{L}\tau_{\vartheta }W_{\vartheta }Q_{\vartheta_{t}}c_{x_{\vartheta }}\right)\; & \left(\sum_{\vartheta =1}^{L}\tau_{\vartheta }W_{\vartheta }Q_{\vartheta_{t}}\right)\\ \left(\sum_{\vartheta =1}^{L}\tau_{\vartheta }W_{\vartheta }c_{x_{\vartheta }}Q_{\vartheta_{t}}\right)\; & \left(\sum_{\vartheta =1}^{L}\tau_{\vartheta }W_{\vartheta }c_{x_{\vartheta }}^{2}\right)\; & \left(\sum_{\vartheta =1}^{L}\tau_{\vartheta }W_{\vartheta }c_{x_{\vartheta }}\right)\\ \left(\sum_{\vartheta =1}^{L}\tau_{\vartheta }W_{\vartheta }Q_{\vartheta_{t}}\right)\; & \left(\sum_{\vartheta =1}^{L}\tau_{\vartheta }W_{\vartheta }c_{x_{\vartheta }}\right)\; & \left(\sum_{\vartheta =1}^{L}\tau_{\vartheta }W_{\vartheta }\right) \end{array}\right].$$

By solving equation (7), we get

$$\eta_{1(\vartheta )}=\frac{D_{1(\vartheta )}}{H},\;\eta_{2(\vartheta )}=\frac{D_{2(\vartheta )}}{H},\;\eta_{3(\vartheta )}=\frac{D_{3(\vartheta )}}{H}$$

where

$$D_{1(\vartheta )}=\sum_{\vartheta =1}^{L}W_{\vartheta }\left({\overset{¯}{X}}_{\vartheta }-Q_{\vartheta_{t}}\right)\left(\sum_{\vartheta =1}^{L}\tau_{\vartheta }W_{\vartheta }\right)\left(\sum_{\vartheta =1}^{L}\tau_{\vartheta }W_{\vartheta }c_{x_{\vartheta }}^{2}\right)-\sum_{\vartheta =1}^{L}W_{\vartheta }\left({\overset{¯}{X}}_{\vartheta }-Q_{\vartheta_{t}}\right){\left(\sum_{\vartheta =1}^{L}\tau_{\vartheta }W_{\vartheta }c_{x_{\vartheta }}\right)}^{2}+\sum_{\vartheta =1}^{L}W_{\vartheta }\left(C_{x_{\vartheta }}-c_{x_{\vartheta }}\right)\left(\sum_{\vartheta =1}^{L}\tau_{\vartheta }W_{\vartheta }Q_{\vartheta_{t}}\right)\left(\sum_{\vartheta =1}^{L}\tau_{\vartheta }W_{\vartheta }c_{x_{\vartheta }}\right)-\sum_{\vartheta =1}^{L}W_{\vartheta }\left(C_{x_{\vartheta }}-c_{x_{\vartheta }}\right)\left(\sum_{\vartheta =1}^{L}\tau_{\vartheta }W_{\vartheta }\right)\left(\sum_{\vartheta =1}^{L}\tau_{\vartheta }W_{\vartheta }c_{x_{\vartheta }}Q_{\vartheta_{t}}\right)$$

$$D_{2(\vartheta )}=\sum_{\vartheta =1}^{L}W_{\vartheta }\left(C_{x_{\vartheta }}-c_{x_{\vartheta }}\right)\left(\sum_{\vartheta =1}^{L}\tau_{\vartheta }W_{\vartheta }\right)\left(\sum_{\vartheta =1}^{L}\tau_{\vartheta }W_{\vartheta }Q_{\vartheta_{t}}^{2}\right)-\sum_{\vartheta =1}^{L}W_{\vartheta }\left(C_{x_{\vartheta }}-c_{x_{\vartheta }}\right){\left(\sum_{\vartheta =1}^{L}\tau_{\vartheta }W_{\vartheta }Q_{\vartheta_{t}}\right)}^{2}-\sum_{\vartheta =1}^{L}W_{\vartheta }\left({\overset{¯}{X}}_{\vartheta }-Q_{\vartheta_{t}}\right)\left(\sum_{\vartheta =1}^{L}\tau_{\vartheta }W_{\vartheta }c_{x_{\vartheta }}Q_{\vartheta_{t}}\right)\left(\sum_{\vartheta =1}^{L}\tau_{\vartheta }W_{\vartheta }\right)+\sum_{\vartheta =1}^{L}W_{\vartheta }\left({\overset{¯}{X}}_{\vartheta }-Q_{\vartheta_{t}}\right)\left(\sum_{\vartheta =1}^{L}\tau_{\vartheta }W_{\vartheta }Q_{\vartheta_{t}}\right)\left(\sum_{\vartheta =1}^{L}\tau_{\vartheta }W_{\vartheta }c_{x_{\vartheta }}\right)$$

$$D_{3(\vartheta )}=\sum_{\vartheta =1}^{L}W_{\vartheta }\left({\overset{¯}{X}}_{\vartheta }-Q_{\vartheta_{t}}\right)\left(\sum_{\vartheta =1}^{L}\tau_{\vartheta }W_{\vartheta }c_{x_{\vartheta }}\right)\left(\sum_{\vartheta =1}^{L}\tau_{\vartheta }W_{\vartheta }c_{x_{\vartheta }}Q_{\vartheta_{t}}\right)-\sum_{\vartheta =1}^{L}W_{\vartheta }\left({\overset{¯}{X}}_{\vartheta }-Q_{\vartheta_{t}}\right)\left(\sum_{\vartheta =1}^{L}\tau_{\vartheta }W_{\vartheta }Q_{\vartheta_{t}}\right)\left(\sum_{\vartheta =1}^{L}\tau_{\vartheta }W_{\vartheta }c_{x_{\vartheta }}^{2}\right)+\sum_{\vartheta =1}^{L}W_{\vartheta }\left(C_{x_{\vartheta }}-c_{x_{\vartheta }}\right)\left(\sum_{\vartheta =1}^{L}\tau_{\vartheta }W_{\vartheta }Q_{\vartheta_{t}}\right)\left(\sum_{\vartheta =1}^{L}\tau_{\vartheta }W_{\vartheta }Q_{\vartheta_{t}}c_{x_{\vartheta }}\right)-\sum_{\vartheta =1}^{L}W_{\vartheta }\left(C_{x_{\vartheta }}-c_{x_{\vartheta }}\right)\left(\sum_{\vartheta =1}^{L}\tau_{\vartheta }W_{\vartheta }Q_{\vartheta_{t}}^{2}\right)\left(\sum_{\vartheta =1}^{L}\tau_{\vartheta }W_{\vartheta }c_{x_{\vartheta }}\right)$$

$$H=\left(\sum_{\vartheta =1}^{L}\tau_{\vartheta }W_{\vartheta }\right)\left(\sum_{\vartheta =1}^{L}\tau_{\vartheta }W_{\vartheta }c_{x_{\vartheta }}^{2}\right)\left(\sum_{\vartheta =1}^{L}\tau_{\vartheta }W_{\vartheta }Q_{\vartheta_{t}}^{2}\right)-{\left(\sum_{\vartheta =1}^{L}\tau_{\vartheta }W_{\vartheta }Q_{\vartheta_{t}}\right)}^{2}\left(\sum_{\vartheta =1}^{L}\tau_{\vartheta }W_{\vartheta }c_{x_{\vartheta }}^{2}\right)-\left(\sum_{\vartheta =1}^{L}\tau_{\vartheta }W_{\vartheta }\right){\left(\sum_{\vartheta =1}^{L}\tau_{\vartheta }W_{\vartheta }c_{x_{\vartheta }}\right)}^{2}-{\left(\sum_{\vartheta =1}^{L}\tau_{\vartheta }W_{\vartheta }c_{x_{\vartheta }}\right)}^{2}\left(\sum_{\vartheta =1}^{L}\tau_{\vartheta }W_{\vartheta }Q_{\vartheta_{t}}^{2}\right)+2\left(\sum_{\vartheta =1}^{L}\tau_{\vartheta }W_{\vartheta }Q_{\vartheta_{t}}\right)\left(\sum_{\vartheta =1}^{L}\tau_{\vartheta }W_{\vartheta }c_{x_{\vartheta }}\right)\left(\sum_{\vartheta =1}^{L}\tau_{\vartheta }W_{\vartheta }Q_{\vartheta_{t}}c_{x_{\vartheta }}\right).$$

Substituting these values in equation (6) and equation (2) and get ${\overset{¯}{y}}_{PM}$ in equation (8) and equation (9)

(8) $${\overset{¯}{y}}_{PM}=\sum_{\vartheta =1}^{L}W_{\vartheta }Z_{\vartheta_{t}}+\eta_{1(\vartheta )}\left(\sum_{\vartheta =1}^{L}\tau_{\vartheta }W_{\vartheta }Q_{\vartheta_{t}}Z_{\vartheta_{t}}\right)+\eta_{2(\vartheta )}\left(\sum_{\vartheta =1}^{L}\tau_{\vartheta }W_{\vartheta }c_{x_{\vartheta }}Z_{\vartheta_{t}}\right)+\eta_{3(\vartheta )}\left(\sum_{\vartheta =1}^{L}\tau_{\vartheta }W_{\vartheta }Z_{\vartheta_{t}}\right)$$

(9) $$=\sum_{\vartheta =1}^{L}W_{\vartheta }Z_{\vartheta_{t}}+{\overset{ˆ}{b}}_{1(j)}\left[\sum_{\vartheta =1}^{L}W_{\vartheta }\left({\overset{¯}{X}}_{\vartheta }-Q_{\vartheta_{t}}\right)\right]+{\overset{ˆ}{b}}_{2(j)}\left[\sum_{\vartheta =1}^{L}W_{\vartheta }\left(C_{x_{\vartheta }}-c_{x_{\vartheta }}\right)\right]$$

where

$${\overset{ˆ}{b}}_{1(j)}=\frac{D_{4(\vartheta )}}{H},\;{\overset{ˆ}{b}}_{2(j)}=\frac{D_{5(\vartheta )}}{H}$$

$$D_{4(\vartheta )}=\left(\sum_{\vartheta =1}^{L}\tau_{\vartheta }W_{\vartheta }Q_{\vartheta_{t}}Z_{\vartheta_{t}}\right)\left(\sum_{\vartheta =1}^{L}\tau_{\vartheta }W_{\vartheta }\right)\left(\sum_{\vartheta =1}^{L}\tau_{\vartheta }W_{\vartheta }c_{x_{\vartheta }}^{2}\right)-\left(\sum_{\vartheta =1}^{L}\tau_{\vartheta }W_{\vartheta }Q_{\vartheta_{t}}Z_{\vartheta_{t}}\right){\left(\sum_{\vartheta =1}^{L}\tau_{\vartheta }W_{\vartheta }c_{x_{\vartheta }}\right)}^{2}-\left(\sum_{\vartheta =1}^{L}\tau_{\vartheta }W_{\vartheta }c_{x_{\vartheta }}Z_{\vartheta_{t}}\right)\left(\sum_{\vartheta =1}^{L}\tau_{\vartheta }W_{\vartheta }c_{x_{\vartheta }}Q_{\vartheta_{t}}\right)\left(\sum_{\vartheta =1}^{L}\tau_{\vartheta }W_{\vartheta }\right)+\left(\sum_{\vartheta =1}^{L}\tau_{\vartheta }W_{\vartheta }c_{x_{\vartheta }}Z_{\vartheta_{t}}\right)\left(\sum_{\vartheta =1}^{L}\tau_{\vartheta }W_{\vartheta }Q_{\vartheta_{t}}\right)\left(\sum_{\vartheta =1}^{L}\tau_{\vartheta }W_{\vartheta }c_{x_{\vartheta }}\right)+\left(\sum_{\vartheta =1}^{L}\tau_{\vartheta }W_{\vartheta }Z_{\vartheta_{t}}\right)\left(\sum_{\vartheta =1}^{L}\tau_{\vartheta }W_{\vartheta }c_{x_{\vartheta }}\right)\left(\sum_{\vartheta =1}^{L}\tau_{\vartheta }W_{\vartheta }c_{x_{\vartheta }}Q_{\vartheta_{t}}\right)-\left(\sum_{\vartheta =1}^{L}\tau_{\vartheta }W_{\vartheta }Z_{\vartheta_{t}}\right)\left(\sum_{\vartheta =1}^{L}\tau_{\vartheta }W_{\vartheta }Q_{\vartheta_{t}}\right)\left(\sum_{\vartheta =1}^{L}\tau_{\vartheta }W_{\vartheta }c_{x_{\vartheta }}^{2}\right)$$

$$D_{5(\vartheta )}=\left(\sum_{\vartheta =1}^{L}\tau_{\vartheta }W_{\vartheta }Q_{\vartheta_{t}}Z_{\vartheta_{t}}\right)\left(\sum_{\vartheta =1}^{L}\tau_{\vartheta }W_{\vartheta }Q_{\vartheta_{t}}\right)\left(\sum_{\vartheta =1}^{L}\tau_{\vartheta }W_{\vartheta }c_{x_{\vartheta }}\right)-\left(\sum_{\vartheta =1}^{L}\tau_{\vartheta }W_{\vartheta }Q_{\vartheta_{t}}Z_{\vartheta_{t}}\right)\left(\sum_{\vartheta =1}^{L}\tau_{\vartheta }W_{\vartheta }\right)\left(\sum_{\vartheta =1}^{L}\tau_{\vartheta }W_{\vartheta }c_{x_{\vartheta }}Q_{\vartheta_{t}}\right)+\left(\sum_{\vartheta =1}^{L}\tau_{\vartheta }W_{\vartheta }Z_{\vartheta_{t}}\right)\left(\sum_{\vartheta =1}^{L}\tau_{\vartheta }W_{\vartheta }Q_{\vartheta_{t}}\right)\left(\sum_{\vartheta =1}^{L}\tau_{\vartheta }W_{\vartheta }Q_{\vartheta_{t}}c_{x_{\vartheta }}\right)-\left(\sum_{\vartheta =1}^{L}\tau_{\vartheta }W_{\vartheta }Z_{\vartheta_{t}}\right)\left(\sum_{\vartheta =1}^{L}\tau_{\vartheta }W_{\vartheta }Q_{\vartheta_{t}}^{2}\right)\left(\sum_{\vartheta =1}^{L}\tau_{\vartheta }W_{\vartheta }c_{x_{\vartheta }}\right)+\left(\sum_{\vartheta =1}^{L}\tau_{\vartheta }W_{\vartheta }c_{x_{\vartheta }}Z_{\vartheta_{t}}\right)\left(\sum_{\vartheta =1}^{L}\tau_{\vartheta }W_{\vartheta }\right)\left(\sum_{\vartheta =1}^{L}\tau_{\vartheta }W_{\vartheta }Q_{\vartheta_{t}}^{2}\right)-\left(\sum_{\vartheta =1}^{L}\tau_{\vartheta }W_{\vartheta }c_{x_{\vartheta }}Z_{\vartheta_{t}}\right){\left(\sum_{\vartheta =1}^{L}\tau_{\vartheta }W_{\vartheta }Q_{\vartheta_{t}}\right)}^{2}.$$

## 3 Proposed memory type calibrated estimator

Taking motivation from Koyuncu [31], we propose memory type calibrated mean estimator ${\overset{¯}{y}}_{TM}$ in equation (10) using EWMA under $S_{t}RS$ as given by

(10) $${\overset{¯}{y}}_{TM}=\sum_{\vartheta =1}^{L}\Psi_{\vartheta }Z_{\vartheta_{t}}$$

subject to the constraints

(11) $$\sum_{\vartheta =1}^{L}\Psi_{\vartheta }=\sum_{\vartheta =1}^{L}W_{\vartheta }$$

(12) $$\sum_{\vartheta =1}^{L}\Psi_{\vartheta }Q_{\vartheta_{t}}=\sum_{\vartheta =1}^{L}W_{\vartheta }{\overset{¯}{X}}_{\vartheta }.$$

Defining the Lagrange function with its multipliers $\eta_{4(\vartheta )}$ and $\eta_{5(\vartheta )}$ using equation (11) and equation (12)

$$\Delta_{\left(b\right)}=\sum_{\vartheta =1}^{L}\frac{{\left(\Psi_{\vartheta }-W_{\vartheta }\right)}^{2}}{\tau_{\vartheta }W_{\vartheta }}-2\eta_{4(\vartheta )}\left[\sum_{\vartheta =1}^{L}\Psi_{\vartheta }-\sum_{\vartheta =1}^{L}W_{\vartheta }\right]-2\eta_{5(\vartheta )}\left[\sum_{\vartheta =1}^{L}\Psi_{\vartheta }Q_{\vartheta_{t}}-\sum_{\vartheta =1}^{L}W_{\vartheta }{\overset{¯}{X}}_{\vartheta }\right].$$

By calculating $\frac{\delta \Delta_{\left(b\right)}}{\delta \Psi_{\vartheta }}=0$, we get

(13) $$\Psi_{\vartheta }=W_{\vartheta }+\tau_{\vartheta }W_{\vartheta }\left[\eta_{4(\vartheta )}Q_{\vartheta_{t}}+\eta_{5(\vartheta )}\right].$$

Putting equation (13) in equation (11) and equation (12), we get

$$\eta_{4(\vartheta )}=-\frac{\sum_{\vartheta =1}^{L}W_{\vartheta }\left[{\overset{¯}{X}}_{\vartheta }-Q_{\vartheta_{t}}\right]\left[\sum_{\vartheta =1}^{L}\tau_{\vartheta }W_{\vartheta }Q_{\vartheta_{t}}\right]}{\left[\sum_{\vartheta =1}^{L}\tau_{\vartheta }W_{\vartheta }Q_{\vartheta_{t}}^{2}\right]\left[\sum_{\vartheta =1}^{L}\tau_{\vartheta }W_{\vartheta }\right]-{\left[\sum_{\vartheta =1}^{L}\tau_{\vartheta }W_{\vartheta }Q_{\vartheta_{t}}\right]}^{2}}$$

$$\eta_{5(\vartheta )}=\frac{\sum_{\vartheta =1}^{L}W_{\vartheta }\left[{\overset{¯}{X}}_{\vartheta }-Q_{\vartheta_{t}}\right]\left[\sum_{\vartheta =1}^{L}\tau_{\vartheta }W_{\vartheta }\right]}{\left[\sum_{\vartheta =1}^{L}\tau_{\vartheta }W_{\vartheta }Q_{\vartheta_{t}}^{2}\right]\left[\sum_{\vartheta =1}^{L}\tau_{\vartheta }W_{\vartheta }\right]-{\left[\sum_{\vartheta =1}^{L}\tau_{\vartheta }W_{\vartheta }Q_{\vartheta_{t}}\right]}^{2}}.$$

By substituting $\eta_{4(\vartheta )}$ and $\eta_{5(\vartheta )}$ in equation (13), we get

$$\Psi_{\vartheta }=W_{\vartheta }+\tau_{\vartheta }W_{\vartheta }\frac{\left[\sum_{\vartheta =1}^{L}\tau_{\vartheta }W_{\vartheta }\right]Q_{\vartheta_{t}}-\left[\sum_{\vartheta =1}^{L}\tau_{\vartheta }W_{\vartheta }Q_{\vartheta_{t}}\right]}{\left[\sum_{\vartheta =1}^{L}\tau_{\vartheta }W_{\vartheta }Q_{\vartheta_{t}}^{2}\right]\left[\sum_{\vartheta =1}^{L}\tau_{\vartheta }W_{\vartheta }\right]-{\left[\sum_{\vartheta =1}^{L}\tau_{\vartheta }W_{\vartheta }Q_{\vartheta_{t}}\right]}^{2}}\sum_{\vartheta =1}^{L}W_{\vartheta }\left[{\overset{¯}{X}}_{\vartheta }-Q_{\vartheta_{t}}\right].$$

By putting $\Psi_{\vartheta }$ in ${\overset{¯}{y}}_{TM}$ and get calibrated mean estimator of study variable

$${\overset{¯}{y}}_{TM}=\sum_{\vartheta =1}^{L}W_{\vartheta }Z_{\vartheta_{t}}+\frac{\left[\sum_{\vartheta =1}^{L}\tau_{\vartheta }W_{\vartheta }\right]\left[\sum_{\vartheta =1}^{L}\tau_{\vartheta }W_{\vartheta }Z_{\vartheta_{t}}Q_{\vartheta_{t}}\right]-\left[\sum_{\vartheta =1}^{L}\tau_{\vartheta }W_{\vartheta }Q_{\vartheta_{t}}\right]\left[\sum_{\vartheta =1}^{L}\tau_{\vartheta }W_{\vartheta }Z_{\vartheta_{t}}\right]}{\left[\sum_{\vartheta =1}^{L}\tau_{\vartheta }W_{\vartheta }Q_{\vartheta_{t}}^{2}\right]\left[\sum_{\vartheta =1}^{L}\tau_{\vartheta }W_{\vartheta }\right]-{\left[\sum_{\vartheta =1}^{L}\tau_{\vartheta }W_{\vartheta }Q_{\vartheta_{t}}\right]}^{2}}\sum_{\vartheta =1}^{L}W_{\vartheta }\left[{\overset{¯}{X}}_{\vartheta }-Q_{\vartheta_{t}}\right].$$

This estimator can be rewritten as

$${\overset{¯}{y}}_{TM}=\sum_{\vartheta =1}^{L}W_{\vartheta }Z_{\vartheta_{t}}+{\overset{ˆ}{b}}_{j1}\left[\sum_{\vartheta =1}^{L}W_{\vartheta }({\overset{¯}{X}}_{\vartheta }-Q_{\vartheta_{t}})\right]$$

where

$${\overset{ˆ}{b}}_{j1}=\frac{\left[\sum_{\vartheta =1}^{L}\tau_{\vartheta }W_{\vartheta }\right]\left[\sum_{\vartheta =1}^{L}\tau_{\vartheta }W_{\vartheta }Z_{\vartheta_{t}}Q_{\vartheta_{t}}\right]-\left[\sum_{\vartheta =1}^{L}\tau_{\vartheta }W_{\vartheta }Q_{\vartheta_{t}}\right]\left[\sum_{\vartheta =1}^{L}\tau_{\vartheta }W_{\vartheta }Z_{\vartheta_{t}}\right]}{\left[\sum_{\vartheta =1}^{L}\tau_{\vartheta }W_{\vartheta }Q_{\vartheta_{t}}^{2}\right]\left[\sum_{\vartheta =1}^{L}\tau_{\vartheta }W_{\vartheta }\right]-{\left[\sum_{\vartheta =1}^{L}\tau_{\vartheta }W_{\vartheta }Q_{\vartheta_{t}}\right]}^{2}}.$$

Note that the references [32], [34] defined many estimators using traditional and non-traditional known population measures. We adapt their work using EWMA frame work.

## 4 Double stratified sampling

This section primarily focuses on case where mean of the auxiliary variable is unknown. For such scenario, our approach extends the framework initially proposed by Shahzad et al. [32] and Koc et al. [7] for the double stratified random sampling design ($S_{t}RS$). However, we modify their framework using calibration techniques. It is important to note that in the $\vartheta^{th}$ stratum, $n_{1\vartheta }$ represents the sample size for the first phase, and $n_{2\vartheta }$ represents the sample size for the second phase. Let $\left(Q_{1\vartheta_{t}},c_{1x_{\vartheta }}\right)$ be the EWMA statistic and coefficient of variation *X* for the first phase. $\left(Q_{2\vartheta_{t}},c_{2x_{\vartheta }}\right)$ be the EWMA statistic and coefficient of variation *X* for the second phase. $Z_{2\vartheta_{t}}$ be the EWMA statistic of *Y* for the second phase in the $\vartheta^{th}$ stratum.

### 4.1 Adapted double memory type calibrated estimator

The adapted estimator ${\overset{¯}{y}}_{PaM}$ under double $S_{t}RS$ as given below in equation (14)

(14) $${\overset{¯}{y}}_{PaM}=\sum_{\vartheta =1}^{L}\Psi_{a\vartheta }Z_{2\vartheta_{t}}$$

subject to the constraints

(15) $$\sum_{\vartheta =1}^{L}\Psi_{a\vartheta }Q_{2\vartheta_{t}}=\sum_{\vartheta =1}^{L}W_{\vartheta }Q_{1\vartheta_{t}}$$

(16) $$\sum_{\vartheta =1}^{L}\Psi_{a\vartheta }c_{2x_{\vartheta }}=\sum_{\vartheta =1}^{L}W_{\vartheta }c_{1x_{\vartheta }}$$

(17) $$\sum_{\vartheta =1}^{L}\Psi_{a\vartheta }=\sum_{\vartheta =1}^{L}W_{\vartheta }.$$

Defining the Lagrange function using equation (15), equation (16) and equation (17) with its multipliers $\eta_{6(\vartheta )}$, $\eta_{7(\vartheta )}$ and $\eta_{8(\vartheta )}$

$$\Delta_{\left(c\right)}=\sum_{\vartheta =1}^{L}\frac{{\left(\Psi_{a\vartheta }-W_{\vartheta }\right)}^{2}}{\tau_{\vartheta }W_{\vartheta }}-2\eta_{6(\vartheta )}\left[\;\sum_{\vartheta =1}^{L}\Psi_{a\vartheta }Q_{2\vartheta_{t}}-\sum_{\vartheta =1}^{L}W_{\vartheta }Q_{1\vartheta_{t}}\;\right]-2\eta_{7(\vartheta )}\left[\sum_{\vartheta =1}^{L}\Psi_{a\vartheta }c_{2x_{\vartheta }}-\sum_{\vartheta =1}^{L}W_{\vartheta }c_{1x_{\vartheta }}\right]-2\eta_{8(\vartheta )}\left[\sum_{\vartheta =1}^{L}\Psi_{a\vartheta }-\sum_{\vartheta =1}^{L}W_{\vartheta }\right].$$

By calculating $\frac{\delta \Delta_{\left(c\right)}}{\delta \Psi_{a\vartheta }}=0$, we get

(18) $$\Psi_{a\vartheta }=W_{\vartheta }+\tau_{\vartheta }W_{\vartheta }\left[\eta_{6(\vartheta )}Q_{2\vartheta_{t}}+\eta_{7(\vartheta )}c_{2x_{\vartheta }}+\eta_{8(\vartheta )}\right].$$

Substituting equation (18) in equation (15), equation (16), and equation (17), respectively, and get

(19) $$G_{\left(3\times 3\right)}\eta_{\left(3\times 1\right)}=F_{\left(3\times 1\right)}$$

where

$$\eta_{\left(3\times 1\right)}=\left[\begin{array}{c} \eta_{6(\vartheta )}\\ \eta_{7(\vartheta )}\\ \eta_{8(\vartheta )} \end{array}\right],$$

$$F_{\left(3\times 1\right)}=\left[\begin{array}{c} \sum_{\vartheta =1}^{L}W_{\vartheta }\left(Q_{1\vartheta_{t}}-Q_{2\vartheta_{t}}\right)\\ \sum_{\vartheta =1}^{L}W_{\vartheta }\left(c_{1x_{\vartheta }}-c_{2x_{\vartheta }}\right)\\ 0 \end{array}\right],$$

$$G_{\left(3\times 3\right)}=\left[\begin{array}{ccc} \left(\sum_{\vartheta =1}^{L}\tau_{\vartheta }W_{\vartheta }Q_{2\vartheta_{t}}^{2}\right)\; & \left(\sum_{\vartheta =1}^{L}\tau_{\vartheta }W_{\vartheta }Q_{2\vartheta_{t}}c_{2x_{\vartheta }}\right)\; & \left(\sum_{\vartheta =1}^{L}\tau_{\vartheta }W_{\vartheta }Q_{2\vartheta_{t}}\right)\\ \left(\sum_{\vartheta =1}^{L}\tau_{\vartheta }W_{\vartheta }c_{2x_{\vartheta }}Q_{2\vartheta_{t}}\right)\; & \left(\sum_{\vartheta =1}^{L}\tau_{\vartheta }W_{\vartheta }c_{2x_{\vartheta }}^{2}\right)\; & \left(\sum_{\vartheta =1}^{L}\tau_{\vartheta }W_{\vartheta }c_{2x_{\vartheta }}\right)\\ \left(\sum_{\vartheta =1}^{L}\tau_{\vartheta }W_{\vartheta }Q_{2\vartheta_{t}}\right)\; & \left(\sum_{\vartheta =1}^{L}\tau_{\vartheta }W_{\vartheta }c_{2x_{\vartheta }}\right)\; & \left(\sum_{\vartheta =1}^{L}\tau_{\vartheta }W_{\vartheta }\right) \end{array}\right].$$

By solving equation (19), we get

$$\eta_{6(\vartheta )}=\frac{D_{6(\vartheta )}}{H_{1}},\;\eta_{7(\vartheta )}=\frac{D_{7(\vartheta )}}{H_{1}},\;\eta_{8(\vartheta )}=\frac{D_{8(\vartheta )}}{H_{1}}$$

where

$$D_{6(\vartheta )}=\sum_{\vartheta =1}^{L}W_{\vartheta }\left(Q_{1\vartheta_{t}}-Q_{2\vartheta_{t}}\right)\left(\sum_{\vartheta =1}^{L}\tau_{\vartheta }W_{\vartheta }\right)\left(\sum_{\vartheta =1}^{L}\tau_{\vartheta }W_{\vartheta }c_{2x_{\vartheta }}^{2}\right)-\sum_{\vartheta =1}^{L}W_{\vartheta }\left(Q_{1\vartheta_{t}}-Q_{2\vartheta_{t}}\right){\left(\sum_{\vartheta =1}^{L}\tau_{\vartheta }W_{\vartheta }c_{2x_{\vartheta }}\right)}^{2}+\sum_{\vartheta =1}^{L}W_{\vartheta }\left(c_{1x_{\vartheta }}-c_{2x_{\vartheta }}\right)\left(\sum_{\vartheta =1}^{L}\tau_{\vartheta }W_{\vartheta }Q_{2\vartheta_{t}}\right)\left(\sum_{\vartheta =1}^{L}\tau_{\vartheta }W_{\vartheta }c_{2x_{\vartheta }}\right)-\sum_{\vartheta =1}^{L}W_{\vartheta }\left(c_{1x_{\vartheta }}-c_{2x_{\vartheta }}\right)\left(\sum_{\vartheta =1}^{L}\tau_{\vartheta }W_{\vartheta }\right)\left(\sum_{\vartheta =1}^{L}\tau_{\vartheta }W_{\vartheta }c_{2x_{\vartheta }}Q_{2\vartheta_{t}}\right)$$

$$D_{7(\vartheta )}=\sum_{\vartheta =1}^{L}W_{\vartheta }\left(c_{1x_{\vartheta }}-c_{2x_{\vartheta }}\right)\left(\sum_{\vartheta =1}^{L}\tau_{\vartheta }W_{\vartheta }\right)\left(\sum_{\vartheta =1}^{L}\tau_{\vartheta }W_{\vartheta }Q_{2\vartheta_{t}}^{2}\right)-\sum_{\vartheta =1}^{L}W_{\vartheta }\left(c_{1x_{\vartheta }}-c_{2x_{\vartheta }}\right){\left(\sum_{\vartheta =1}^{L}\tau_{\vartheta }W_{\vartheta }Q_{2\vartheta_{t}}\right)}^{2}-\sum_{\vartheta =1}^{L}W_{\vartheta }\left(Q_{1\vartheta_{t}}-Q_{2\vartheta_{t}}\right)\left(\sum_{\vartheta =1}^{L}\tau_{\vartheta }W_{\vartheta }c_{2x_{\vartheta }}Q_{2\vartheta_{t}}\right)\left(\sum_{\vartheta =1}^{L}\tau_{\vartheta }W_{\vartheta }\right)+\sum_{\vartheta =1}^{L}W_{\vartheta }\left(Q_{1\vartheta_{t}}-Q_{2\vartheta_{t}}\right)\left(\sum_{\vartheta =1}^{L}\tau_{\vartheta }W_{\vartheta }Q_{2\vartheta_{t}}\right)\left(\sum_{\vartheta =1}^{L}\tau_{\vartheta }W_{\vartheta }c_{2x_{\vartheta }}\right)$$

$$D_{8(\vartheta )}=\sum_{\vartheta =1}^{L}W_{\vartheta }\left(Q_{1\vartheta_{t}}-Q_{2\vartheta_{t}}\right)\left(\sum_{\vartheta =1}^{L}\tau_{\vartheta }W_{\vartheta }c_{2x_{\vartheta }}\right)\left(\sum_{\vartheta =1}^{L}\tau_{\vartheta }W_{\vartheta }c_{2x_{\vartheta }}Q_{2\vartheta_{t}}\right)-\sum_{\vartheta =1}^{L}W_{\vartheta }\left(Q_{1\vartheta_{t}}-Q_{2\vartheta_{t}}\right)\left(\sum_{\vartheta =1}^{L}\tau_{\vartheta }W_{\vartheta }Q_{2\vartheta_{t}}\right)\left(\sum_{\vartheta =1}^{L}\tau_{\vartheta }W_{\vartheta }c_{2x_{\vartheta }}^{2}\right)+\sum_{\vartheta =1}^{L}W_{\vartheta }\left(c_{1x_{\vartheta }}-c_{2x_{\vartheta }}\right)\left(\sum_{\vartheta =1}^{L}\tau_{\vartheta }W_{\vartheta }Q_{2\vartheta_{t}}\right)\left(\sum_{\vartheta =1}^{L}\tau_{\vartheta }W_{\vartheta }Q_{2\vartheta_{t}}c_{2x_{\vartheta }}\right)-\sum_{\vartheta =1}^{L}W_{\vartheta }\left(c_{1x_{\vartheta }}-c_{2x_{\vartheta }}\right)\left(\sum_{\vartheta =1}^{L}\tau_{\vartheta }W_{\vartheta }Q_{2\vartheta_{t}}^{2}\right)\left(\sum_{\vartheta =1}^{L}\tau_{\vartheta }W_{\vartheta }c_{2x_{\vartheta }}\right)$$

$$H_{1}=\left(\sum_{\vartheta =1}^{L}\tau_{\vartheta }W_{\vartheta }\right)\left(\sum_{\vartheta =1}^{L}\tau_{\vartheta }W_{\vartheta }c_{2x_{\vartheta }}^{2}\right)\left(\sum_{\vartheta =1}^{L}\tau_{\vartheta }W_{\vartheta }Q_{2\vartheta_{t}}^{2}\right)-{\left(\sum_{\vartheta =1}^{L}\tau_{\vartheta }W_{\vartheta }Q_{2\vartheta_{t}}\right)}^{2}\left(\sum_{\vartheta =1}^{L}\tau_{\vartheta }W_{\vartheta }c_{2x_{\vartheta }}^{2}\right)-\left(\sum_{\vartheta =1}^{L}\tau_{\vartheta }W_{\vartheta }\right){\left(\sum_{\vartheta =1}^{L}\tau_{\vartheta }W_{\vartheta }c_{2x_{\vartheta }}\right)}^{2}-{\left(\sum_{\vartheta =1}^{L}\tau_{\vartheta }W_{\vartheta }c_{2x_{\vartheta }}\right)}^{2}\left(\sum_{\vartheta =1}^{L}\tau_{\vartheta }W_{\vartheta }Q_{2\vartheta_{t}}^{2}\right)+2\left(\sum_{\vartheta =1}^{L}\tau_{\vartheta }W_{\vartheta }Q_{2\vartheta_{t}}\right)\left(\sum_{\vartheta =1}^{L}\tau_{\vartheta }W_{\vartheta }c_{2x_{\vartheta }}\right)\left(\sum_{\vartheta =1}^{L}\tau_{\vartheta }W_{\vartheta }Q_{2\vartheta_{t}}c_{2x_{\vartheta }}\right).$$

Substituting these values in equation (18) and equation (14), we have

$${\overset{¯}{y}}_{PaM}=\sum_{\vartheta =1}^{L}W_{\vartheta }Z_{2\vartheta_{t}}+\eta_{6(\vartheta )}\left(\sum_{\vartheta =1}^{L}\tau_{\vartheta }W_{\vartheta }Q_{2\vartheta_{t}}Z_{2\vartheta_{t}}\right)+\eta_{7(\vartheta )}\left(\sum_{\vartheta =1}^{L}\tau_{\vartheta }W_{\vartheta }c_{2x_{\vartheta }}Z_{2\vartheta_{t}}\right)+\eta_{8(\vartheta )}\left(\sum_{\vartheta =1}^{L}\tau_{\vartheta }W_{\vartheta }Z_{2\vartheta_{t}}\right),=\sum_{\vartheta =1}^{L}W_{\vartheta }Z_{2\vartheta_{t}}+{\overset{ˆ}{b}}_{3(j)}\left[\sum_{\vartheta =1}^{L}W_{\vartheta }\left(Q_{1\vartheta_{t}}-Q_{2\vartheta_{t}}\right)\right]+{\overset{ˆ}{b}}_{4(j)}\left[\sum_{\vartheta =1}^{L}W_{\vartheta }\left(c_{1x_{\vartheta }}-c_{2x_{\vartheta }}\right)\right]$$

where

$${\overset{ˆ}{b}}_{3(j)}=\frac{D_{9(\vartheta )}}{H_{1}},\;{\overset{ˆ}{b}}_{4(j)}=\frac{D_{10(\vartheta )}}{H_{1}}$$

$$D_{9(\vartheta )}=\left(\sum_{\vartheta =1}^{L}\tau_{\vartheta }W_{\vartheta }Z_{2\vartheta_{t}}\right)\left(\sum_{\vartheta =1}^{L}\tau_{\vartheta }W_{\vartheta }c_{2x_{\vartheta }}\right)\left(\sum_{\vartheta =1}^{L}\tau_{\vartheta }W_{\vartheta }c_{2x_{\vartheta }}Q_{2\vartheta_{t}}\right)-\left(\sum_{\vartheta =1}^{L}\tau_{\vartheta }W_{\vartheta }Z_{2\vartheta_{t}}\right)\left(\sum_{\vartheta =1}^{L}\tau_{\vartheta }W_{\vartheta }Q_{2\vartheta_{t}}\right)\left(\sum_{\vartheta =1}^{L}\tau_{\vartheta }W_{\vartheta }c_{2x_{\vartheta }}^{2}\right)-\left(\sum_{\vartheta =1}^{L}\tau_{\vartheta }W_{\vartheta }c_{2x_{\vartheta }}Z_{2\vartheta_{t}}\right)\left(\sum_{\vartheta =1}^{L}\tau_{\vartheta }W_{\vartheta }c_{2x_{\vartheta }}Q_{2\vartheta_{t}}\right)\left(\sum_{\vartheta =1}^{L}\tau_{\vartheta }W_{\vartheta }\right)+\left(\sum_{\vartheta =1}^{L}\tau_{\vartheta }W_{\vartheta }c_{2x_{\vartheta }}Z_{2\vartheta_{t}}\right)\left(\sum_{\vartheta =1}^{L}\tau_{\vartheta }W_{\vartheta }Q_{2\vartheta_{t}}\right)\left(\sum_{\vartheta =1}^{L}\tau_{\vartheta }W_{\vartheta }c_{2x_{\vartheta }}\right)+\left(\sum_{\vartheta =1}^{L}\tau_{\vartheta }W_{\vartheta }Q_{2\vartheta_{t}}Z_{2\vartheta_{t}}\right)\left(\sum_{\vartheta =1}^{L}\tau_{\vartheta }W_{\vartheta }\right)\left(\sum_{\vartheta =1}^{L}\tau_{\vartheta }W_{\vartheta }c_{2x_{\vartheta }}^{2}\right)-\left(\sum_{\vartheta =1}^{L}\tau_{\vartheta }W_{\vartheta }Q_{2\vartheta_{t}}Z_{2\vartheta_{t}}\right){\left(\sum_{\vartheta =1}^{L}\tau_{\vartheta }W_{\vartheta }c_{2x_{\vartheta }}\right)}^{2}$$

$$D_{10(\vartheta )}=\left(\sum_{\vartheta =1}^{L}\tau_{\vartheta }W_{\vartheta }Q_{2\vartheta_{t}}Z_{2\vartheta_{t}}\right)\left(\sum_{\vartheta =1}^{L}\tau_{\vartheta }W_{\vartheta }Q_{2\vartheta_{t}}\right)\left(\sum_{\vartheta =1}^{L}\tau_{\vartheta }W_{\vartheta }c_{2x_{\vartheta }}\right)-\left(\sum_{\vartheta =1}^{L}\tau_{\vartheta }W_{\vartheta }Q_{2\vartheta_{t}}Z_{2\vartheta_{t}}\right)\left(\sum_{\vartheta =1}^{L}\tau_{\vartheta }W_{\vartheta }Q_{2\vartheta_{t}}\right)\left(\sum_{\vartheta =1}^{L}\tau_{\vartheta }W_{\vartheta }c_{2x_{\vartheta }}\right)+\left(\sum_{\vartheta =1}^{L}\tau_{\vartheta }W_{\vartheta }Z_{2\vartheta_{t}}\right)\left(\sum_{\vartheta =1}^{L}\tau_{\vartheta }W_{\vartheta }Q_{2\vartheta_{t}}\right)\left(\sum_{\vartheta =1}^{L}\tau_{\vartheta }W_{\vartheta }Q_{2\vartheta_{t}}c_{2x_{\vartheta }}\right)-\left(\sum_{\vartheta =1}^{L}\tau_{\vartheta }W_{\vartheta }Z_{2\vartheta_{t}}\right)\left(\sum_{\vartheta =1}^{L}\tau_{\vartheta }W_{\vartheta }Q_{2\vartheta_{t}}^{2}\right)\left(\sum_{\vartheta =1}^{L}\tau_{\vartheta }W_{\vartheta }c_{2x_{\vartheta }}\right)+\left(\sum_{\vartheta =1}^{L}\tau_{\vartheta }W_{\vartheta }c_{2x_{\vartheta }}Z_{2\vartheta_{t}}\right)\left(\sum_{\vartheta =1}^{L}\tau_{\vartheta }W_{\vartheta }\right)\left(\sum_{\vartheta =1}^{L}\tau_{\vartheta }W_{\vartheta }Q_{2\vartheta_{t}}^{2}\right)-\left(\sum_{\vartheta =1}^{L}\tau_{\vartheta }W_{\vartheta }c_{2x_{\vartheta }}Z_{2\vartheta_{t}}\right){\left(\sum_{\vartheta =1}^{L}\tau_{\vartheta }W_{\vartheta }Q_{2\vartheta_{t}}\right)}^{2}.$$

### 4.2 Proposed double memory type calibrated estimator

The proposed estimator ${\overset{¯}{y}}_{TaM}$ under double $S_{t}RS$ as given below in equation (20)

(20) $${\overset{¯}{y}}_{TaM}=\sum_{\vartheta =1}^{L}\Psi a_{\vartheta }Z_{2\vartheta_{t}}$$

subject to the constraints

(21) $$\sum_{\vartheta =1}^{L}\Psi a_{\vartheta }=\sum_{\vartheta =1}^{L}W_{\vartheta }$$

(22) $$\sum_{\vartheta =1}^{L}\Psi a_{\vartheta }Q_{2\vartheta_{t}}=\sum_{\vartheta =1}^{L}W_{\vartheta }Q_{1\vartheta_{t}}.$$

Defining the Lagrange function using equation (21) and equation (22) with its multipliers $\eta_{9(\vartheta )}$ and $\eta_{10(\vartheta )}$

$$\Delta_{\left(d\right)}=\sum_{\vartheta =1}^{L}\frac{{\left(\Psi a_{\vartheta }-W_{\vartheta }\right)}^{2}}{\tau_{\vartheta }W_{\vartheta }}-2\eta_{9(\vartheta )}\left[\sum_{\vartheta =1}^{L}\Psi a_{\vartheta }-\sum_{\vartheta =1}^{L}W_{\vartheta }\right]-2\eta_{10(\vartheta )}\left[\sum_{\vartheta =1}^{L}\Psi a_{\vartheta }Q_{2\vartheta_{t}}-\sum_{\vartheta =1}^{L}W_{\vartheta }Q_{1\vartheta_{t}}\right].$$

By calculating $\frac{\delta \Delta_{\left(d\right)}}{\delta \Psi_{a\vartheta }}=0$, we get

(23) $$\Psi a_{\vartheta }=W_{\vartheta }+\tau_{\vartheta }W_{\vartheta }\left[\eta_{10(\vartheta )}Q_{2\vartheta_{t}}+\eta_{9(\vartheta )}\right].$$

Putting equation (23) in equation (21) and equation (22), we get

$$\eta_{9(\vartheta )}=-\frac{\sum_{\vartheta =1}^{L}W_{\vartheta }\left[Q_{1\vartheta_{t}}-Q_{2\vartheta_{t}}\right]\left[\sum_{\vartheta =1}^{L}\tau_{\vartheta }W_{\vartheta }Q_{2\vartheta_{t}}\right]}{\left[\sum_{\vartheta =1}^{L}\tau_{\vartheta }W_{\vartheta }Q_{2\vartheta_{t}}^{2}\right]\left[\sum_{\vartheta =1}^{L}\tau_{\vartheta }W_{\vartheta }\right]-{\left[\sum_{\vartheta =1}^{L}\tau_{\vartheta }W_{\vartheta }Q_{2\vartheta_{t}}\right]}^{2}}$$

$$\eta_{10(\vartheta )}=\frac{\sum_{\vartheta =1}^{L}W_{\vartheta }\left[Q_{1\vartheta_{t}}-Q_{2\vartheta_{t}}\right]\left[\sum_{\vartheta =1}^{L}\tau_{\vartheta }W_{\vartheta }\right]}{\left[\sum_{\vartheta =1}^{L}\tau_{\vartheta }W_{\vartheta }Q_{2\vartheta_{t}}^{2}\right]\left[\sum_{\vartheta =1}^{L}\tau_{\vartheta }W_{\vartheta }\right]-{\left[\sum_{\vartheta =1}^{L}\tau_{\vartheta }W_{\vartheta }Q_{2\vartheta_{t}}\right]}^{2}}.$$

By substituting $\eta_{9(\vartheta )}$ and $\eta_{10(\vartheta )}$ in equation (23), we get

$$\Psi a_{\vartheta }=W_{\vartheta }+\tau_{\vartheta }W_{\vartheta }\frac{\left[\sum_{\vartheta =1}^{L}\tau_{\vartheta }W_{\vartheta }\right]Q_{2\vartheta_{t}}-\left[\sum_{\vartheta =1}^{L}\tau_{\vartheta }W_{\vartheta }Q_{2\vartheta_{t}}\right]}{\left[\sum_{\vartheta =1}^{L}\tau_{\vartheta }W_{\vartheta }Q_{2\vartheta_{t}}^{2}\right]\left[\sum_{\vartheta =1}^{L}\tau_{\vartheta }W_{\vartheta }\right]-{\left[\sum_{\vartheta =1}^{L}\tau_{\vartheta }W_{\vartheta }Q_{2\vartheta_{t}}\right]}^{2}}\sum_{\vartheta =1}^{L}W_{\vartheta }\left[Q_{1\vartheta_{t}}-Q_{2\vartheta_{t}}\right].$$

By putting $\Psi a_{\vartheta }$ in ${\overset{¯}{y}}_{TaM}$

$${\overset{¯}{y}}_{TaM}=\sum_{\vartheta =1}^{L}W_{\vartheta }Z_{2\vartheta_{t}}+\frac{\left[\sum_{\vartheta =1}^{L}\tau_{\vartheta }W_{\vartheta }\right]\left[\sum_{\vartheta =1}^{L}\tau_{\vartheta }W_{\vartheta }Z_{2\vartheta_{t}}Q_{2\vartheta_{t}}\right]-\left[\sum_{\vartheta =1}^{L}\tau_{\vartheta }W_{\vartheta }Q_{2\vartheta_{t}}\right]\left[\sum_{\vartheta =1}^{L}\tau_{\vartheta }W_{\vartheta }Z_{2\vartheta_{t}}\right]}{\left[\sum_{\vartheta =1}^{L}\tau_{\vartheta }W_{\vartheta }Q_{2\vartheta_{t}}^{2}\right]\left[\sum_{\vartheta =1}^{L}\tau_{\vartheta }W_{\vartheta }\right]-{\left[\sum_{\vartheta =1}^{L}\tau_{\vartheta }W_{\vartheta }Q_{2\vartheta_{t}}\right]}^{2}}\sum_{\vartheta =1}^{L}W_{\vartheta }\left[Q_{1\vartheta_{t}}-Q_{2\vartheta_{t}}\right].$$

This estimator can be rewritten as

$${\overset{¯}{y}}_{TaM}=\sum_{\vartheta =1}^{L}W_{\vartheta }Z_{2\vartheta_{t}}+{\overset{ˆ}{b}}_{j}\left[\sum_{\vartheta =1}^{L}W_{\vartheta }(Q_{1\vartheta_{t}}-Q_{2\vartheta_{t}})\right]$$

where

$${\overset{ˆ}{b}}_{j}=\frac{\left[\sum_{\vartheta =1}^{L}\tau_{\vartheta }W_{\vartheta }\right]\left[\sum_{\vartheta =1}^{L}\tau_{\vartheta }W_{\vartheta }Z_{2\vartheta_{t}}Q_{2\vartheta_{t}}\right]-\left[\sum_{\vartheta =1}^{L}\tau_{\vartheta }W_{\vartheta }Q_{2\vartheta_{t}}\right]\left[\sum_{\vartheta =1}^{L}\tau_{\vartheta }W_{\vartheta }Z_{2\vartheta_{t}}\right]}{\left[\sum_{\vartheta =1}^{L}\tau_{\vartheta }W_{\vartheta }Q_{2\vartheta_{t}}^{2}\right]\left[\sum_{\vartheta =1}^{L}\tau_{\vartheta }W_{\vartheta }\right]-{\left[\sum_{\vartheta =1}^{L}\tau_{\vartheta }W_{\vartheta }Q_{2\vartheta_{t}}\right]}^{2}}.$$

## 5 Simulation study with diverse applications

To ensure a equitable comparison between the modified estimators $({\overset{¯}{y}}_{PM}$, ${\overset{¯}{y}}_{PaM})$ and the proposed ones $({\overset{¯}{y}}_{TM}$, ${\overset{¯}{y}}_{TaM})$, we acquire varied samples (5%, 10%, 15%) within the $S_{t}RS$ framework for each corresponding stratum in both the initial and subsequent stages.

We select $R_{b}=5000$, the number of samples under $S_{t}RS$ in single-stage and double-stage frame work. For each sample, we calculated the estimates of the population mean $\mu_{y}$. The expressions for (MSE) and (PRE) are

$$\text{MSE}({\overset{¯}{y}}_{PM})=\sum_{k_{b}=1}^{R_{b}}{\left({\overset{¯}{y}}_{PM}-\mu_{y}\right)}^{2}/R_{b}$$

$$\text{MSE}({\overset{¯}{y}}_{TM})=\sum_{k_{b}=1}^{R_{b}}{\left({\overset{¯}{y}}_{TM}-\mu_{y}\right)}^{2}/R_{b}$$

$$\text{MSE}({\overset{¯}{y}}_{PaM})=\sum_{k_{b}=1}^{R_{b}}{\left({\overset{¯}{y}}_{PaM}-\mu_{y}\right)}^{2}/R_{b}$$

$$\text{MSE}(({\overset{¯}{y}}_{TaM})=\sum_{k_{b}=1}^{R_{b}}({\left({\overset{¯}{y}}_{TaM}-\mu_{y}\right)}^{2}/R_{b}$$

$$\text{PRE}({\overset{¯}{y}}_{PM},{\overset{¯}{y}}_{TM})=\frac{\text{MSE}({\overset{¯}{y}}_{PM})}{\text{MSE}({\overset{¯}{y}}_{TM})}\times 100$$

$$\text{PRE}({\overset{¯}{y}}_{PaM},{\overset{¯}{y}}_{TaM})=\frac{\text{MSE}({\overset{¯}{y}}_{PaM})}{\text{MSE}({\overset{¯}{y}}_{TaM})}\times 100.$$

### 5.1 Stock Market data (Poplulation-1)

In this section, we have assessed the estimators using the time scaled data of two registered banks (HBL and UBL) in Islamabad Stock Market. HBL is known as Habib Bank Limited and UBL is known as United Bank Limited. The average of opening and closing quotations from January 2019 to November 2019 daily basis data is taken as X. The average of opening and closing quotations from January 2020 to November 2020 daily basis (time series) data is taken as Y. We find the MSE and PRE of each estimator. It is worth mentioning that HBL is considered as stratum-I and UBL is considered as stratum-II. For more details about data, see references [33], [34]. Some key characteristics are:

• **Stratum-I**$N_{\vartheta 1}=218$**,**$n_{\vartheta 1}=11$**,**$\mu_{y_{\vartheta 1}}=127.68$**,**$\mu_{x_{\vartheta 1}}=127.93$**,**$C_{x_{\vartheta 1}}=0.075$**,**$C_{y_{\vartheta 1}}=0.184$**.**
• **Stratum-II**$N_{\vartheta 2}=218$**,**$n_{\vartheta 2}=11$**,**$\mu_{y_{\vartheta 2}}=126.79$**,**$\mu_{x_{\vartheta 2}}=143.03$**,**$C_{x_{\vartheta 2}}=0.056$**,**$C_{y_{\vartheta 2}}=0.205$**.**

### 5.2 Temperature data (Poplulation-2)

We have considered daily basis (time series) data for the two cities (Lahore and Karachi) of pakistan as population-2. The temperature for 2021 is taken as X. The temperature for 2022 is taken as Y. It is worth mentioning that Karachi is considered as stratum-I and Lahore is considered as stratum-II. Data is collected from publicly available website (https://www.wunderground.com, accessed on 10 June 2023). Some key characteristics are: ${\overset{¯}{X}}_{\vartheta }$

• **Stratum-I**$N_{\vartheta 1}=365$**,**$\mu_{y_{\vartheta 1}}=90.09$**,**$\mu_{x_{\vartheta 1}}=90.82$**,**$C_{x_{\vartheta 1}}=0.080$**,**$C_{y_{\vartheta 1}}=0.082$**.**
• **Stratum-II**$N_{\vartheta 2}=365$**,**$\mu_{y_{\vartheta 2}}=90.82$**,**$\mu_{x_{\vartheta 2}}=86.09$**,**$C_{x_{\vartheta 2}}=0.155$**,**$C_{y_{\vartheta 2}}=0.174$**.**

Note that in population 1 and 2, $\left(N_{\vartheta 1},N_{\vartheta 2}\right)$ are strata sizes, $\left(C_{x_{\vartheta 1}},C_{x_{\vartheta 2}},C_{y_{\vartheta 1}},C_{y_{\vartheta 2}}\right)$ are the coefficient of variations, and $(\mu_{x_{\vartheta 1}},\mu_{x_{\vartheta 2}},\mu_{y_{\vartheta 1}},\mu_{y_{\vartheta 2}}$ are the means of study and auxiliary variables, respectively.

### 5.3 Symmetric data (Poplulation-3)

We generate bi-variate symmetric data for two strata using (Gaussian) distributions. The size of each stratum is 1000. The Gaussian distributions were characterized by $\left(\mu_{x}=2.1,\mu_{y}=3.8\right)$, with variance-covariance matrices specified as follows:

• Stratum 1
  $$\Sigma_{xy}=\left[\begin{array}{cc} 1\; & 0.81\\ 0.81\; & 1 \end{array}\right]$$
• Stratum 2
  $$\Sigma_{xy}=\left[\begin{array}{cc} 1\; & 0.68\\ 0.68\; & 1 \end{array}\right]$$

### 5.4 Results discussion

The results for diverse populations are presented in Table 1, Table 2, Table 3, which display the following key findings:

• Table 1 shows the results of complete information. Here minimum MSE values of adapted estimator ${\overset{¯}{y}}_{PM}$ for populations 1, 2 and 3 at λ=0.05 are (1.0083,0.9907,0.9879), respectively. Similarly, minimum MSE values of proposed estimator ${\overset{¯}{y}}_{TM}$ for populations 1, 2 and 3 at λ=0.05 are (0.0204,0.0028,0.2978), respectively.
• Table 2 shows the results of partial information when mean of auxiliary variable is unknown and we move towards double sampling, as described in Section 4. In Table 2 minimum MSE values of adapted estimator ${\overset{¯}{y}}_{PM}$ for populations 1, 2 and 3 at λ=0.05 are (1.0077,0.9907,0.8998), respectively. Similarly, minimum MSE values of proposed estimator ${\overset{¯}{y}}_{TM}$ for populations 1, 2 and 3 at λ=0.05 are (0.0198,0.0028,0.2878), respectively.
• As shown in Table 3, it's clear that the PRE values exceed 100 for all the proposed estimators. Similar MSE based superiority of proposed estimators can be seen in Table 1, Table 2, as discussed in above points.

**Table 1:** MSE using complete information.

	*λ*	Adapted estimator			Proposed estimator		
		${\overset{¯}{y}}_{PM}$ 5%	${\overset{¯}{y}}_{PM}$ 10%	${\overset{¯}{y}}_{PM}$ 15%	${\overset{¯}{y}}_{TM}$ 5%	${\overset{¯}{y}}_{TM}$ 10%	${\overset{¯}{y}}_{TM}$ 15%
Population-1							
	0.05	1.0530	1.0194	1.0083	0.0651	0.0315	0.0204
	0.10	1.2483	1.1139	1.0698	0.2604	0.1260	0.0819
	0.25	2.6159	1.7758	1.4998	1.6280	0.7879	0.5119
	0.50	7.4999	4.1395	3.0355	6.5120	3.1516	2.0476
	0.75	15.640	8.0791	5.5950	14.6521	7.0912	4.6071
	1.00	27.036	13.594	9.1784	26.0482	12.6066	8.1905
Population-2							
	0.05	0.9970	0.9924	0.9907	0.0091	0.0045	0.0028
	0.10	1.0245	1.0059	0.9992	0.0366	0.0180	0.0113
	0.25	1.2166	1.1005	1.0589	0.2287	0.1126	0.0710
	0.50	1.9028	1.4385	1.2721	0.9149	0.4506	0.2842
	0.75	3.0465	2.0017	1.6274	2.0586	1.0138	0.6395
	1.00	4.6476	2.7903	2.1248	3.6597	1.8024	1.1369
Population-3							
	0.05	1.3866	0.9879	0.9998	0.7974	0.3978	0.2978
	0.10	1.3867	0.9879	0.9999	0.7975	0.3978	0.2978
	0.25	1.3873	0.9882	1.0003	0.7981	0.3981	0.2980
	0.50	1.3896	0.9893	1.0007	0.8004	0.3992	0.2987
	0.75	1.3935	0.9912	1.0019	0.8043	0.4011	0.2999
	1.00	1.3989	0.9938	1.0035	0.8097	0.4037	0.3015

**Table 2:** MSE using partial information.

	*λ*	Adapted estimator			Proposed estimator		
		${\overset{¯}{y}}_{PaM}$ 5%	${\overset{¯}{y}}_{PaM}$ 10%	${\overset{¯}{y}}_{PaM}$ 15%	${\overset{¯}{y}}_{TaM}$ 5%	${\overset{¯}{y}}_{TaM}$ 10%	${\overset{¯}{y}}_{TaM}$ 15%
Population-1							
	0.05	1.0543	1.0196	1.0077	0.0664	0.0317	0.0198
	0.10	1.2538	1.1149	1.0671	0.2659	0.1270	0.0792
	0.25	2.6499	1.7818	1.4834	1.6620	0.7939	0.4955
	0.50	7.6362	4.1637	2.9702	6.6483	3.1758	1.9823
	0.75	15.946	8.1336	5.4481	14.958	7.1457	4.4602
	1.00	27.581	13.691	8.9171	26.593	12.703	7.9292
Population-2							
	0.05	0.9969	0.9922	0.9907	0.0090	0.0043	0.0028
	0.10	1.0240	1.0054	0.9991	0.0361	0.0175	0.0112
	0.25	1.2135	1.0974	1.0580	0.2256	0.1095	0.0701
	0.50	1.8906	1.4258	1.2684	0.9027	0.4379	0.2805
	0.75	3.0189	1.9733	1.6191	2.0310	0.9854	0.6312
	1.00	4.5985	2.7398	2.1101	3.6106	1.7519	1.1222
Population-3							
	0.05	0.9879	1.1877	0.8998	0.3987	0.5996	0.2878
	0.10	0.9880	1.1876	0.8998	0.3988	0.5996	0.2878
	0.25	0.9887	1.1880	0.9005	0.3995	0.5999	0.2880
	0.50	0.9912	1.1892	0.9008	0.4020	0.6011	0.2888
	0.75	0.9953	1.1912	0.9020	0.4061	0.6031	0.2901
	1.00	1.0011	1.1940	0.9038	0.4119	0.6059	0.2918

**Table 3:** PRE of proposed estimators.

	*λ*	Using complete information			Using partial information		
		5%	10%	15%	5%	10%	15%
Population-1							
	0.05	1617.01	3234.50	4924.52	1585.92	3210.60	5083.49
	0.10	479.25	883.63	1306.14	471.48	877.65	1345.88
	0.25	160.68	225.38	292.98	159.43	224.42	299.34
	0.50	115.17	131.34	148.24	114.85	131.10	149.83
	0.75	106.74	113.93	121.44	106.60	113.82	122.14
	1.00	103.79	107.83	112.06	103.71	107.77	112.45
Population-2							
	0.05	10889.22	22008.93	34832.10	11036.11	22640.18	35286.68
	0.10	2798.38	5579.21	8786.05	2835.08	5737.10	8899.73
	0.25	531.84	976.86	1490.06	537.71	1002.13	1508.25
	0.50	207.97	319.23	447.53	209.43	325.55	452.08
	0.75	147.98	197.43	254.46	148.64	200.24	256.48
	1.00	126.99	154.81	186.88	127.36	156.38	188.02
Population-3							
	0.05	173.88	248.33	335.72	247.76	198.07	312.64
	0.10	173.87	248.31	335.69	247.73	198.07	312.61
	0.25	173.81	248.20	335.54	247.47	198.01	312.46
	0.50	173.60	247.78	334.98	246.56	197.82	311.90
	0.75	173.25	247.10	334.07	245.06	197.50	310.97
	1.00	172.76	246.15	332.79	243.02	197.05	309.69

## 6 Conclusion

To enhance the performance of estimators in time-scaled surveys, it's necessary to simultaneously incorporate both historical and current sample data. To tackle this challenge, we employed the EWMA statistic and introduced calibrated estimators within the framework of $S_{t}RS$. In this paper, our emphasis lies in customizing the estimators originally put forth by Koyuncu [31] and Shahzad et al. [29] to suit the $S_{t}RS$ design, a well-established sampling technique used across diverse fields. The findings in section 5 demonstrate that the proposed calibrated estimators outperform the adapted estimators in terms of efficiency. Considering the MSE and PRE values, it becomes apparent that the proposed estimators lead to a significant enhancement in the accuracy of population mean estimation for time-scaled surveys. Furthermore, this study holds the potential for extension to various other sampling designs, albeit with limitations such as time-scaled surveys. In future studies, the work can be extended for Hybrid EWMA.

**Funding**

This work was supported by the 10.13039/501100023674Deanship of Scientific Research, Vice Presidency for Graduate Studies and Scientific Research, 10.13039/501100020912King Faisal University, Saudi Arabia [Grant No. 6069].

## CRediT authorship contribution statement

**Abdullah Mohammed Alomair:** Writing – review & editing, Writing – original draft, Visualization, Validation, Supervision, Software, Resources, Project administration, Methodology, Investigation, Funding acquisition, Formal analysis, Data curation, Conceptualization. **Soofia Iftikhar:** Writing – review & editing, Visualization, Validation, Methodology, Investigation, Formal analysis, Data curation, Conceptualization.

## Declaration of Competing Interest

The authors declare that they have no known competing financial interests or personal relationships that could have appeared to influence the work reported in this paper.

## Data Availability

Data will be made available on request.
